# An ongoing secondary task can reduce the illusory truth effect

**DOI:** 10.3389/fpsyg.2023.1215432

**Published:** 2024-01-03

**Authors:** Deva P. Ly, Daniel M. Bernstein, Eryn J. Newman

**Affiliations:** ^1^School of Medicine and Psychology, Australian National University, Canberra, ACT, Australia; ^2^Department of Psychology, Kwantlen Polytechnic University, Surrey, BC, Canada

**Keywords:** illusory truth effect, truth judgment, secondary task, processing fluency, familiarity

## Abstract

**Introduction:**

People are more likely to believe repeated information—this is known as the Illusory Truth Effect (ITE). Recent research on the ITE has shown that semantic processing of statements plays a key role. In our day to day experience, we are often multi-tasking which can impact our ongoing processing of information around us. In three experiments, we investigate how asking participants to engage in an ongoing secondary task in the ITE paradigm influences the magnitude of the effect of repetition on belief.

**Methods:**

Using an adapted ITE paradigm, we embedded a secondary task into each trial of the encoding and/or test phase (e.g., having participants count the number of vowels in a target word of each trivia claim) and calculated the overall accuracy on the task.

**Results:**

We found that the overall ITE was larger when participants had no ongoing secondary task during the experiment. Further, we predicted and found that higher accuracy on the secondary task was associated with a larger ITE.

**Discussion:**

These findings provide initial evidence that engaging in an ongoing secondary task may reduce the impact of repetition. Our findings suggest that exploring the impact of secondary tasks on the ITE is a fruitful area for further research.

## 1 Introduction

With the proliferation of misinformation and concerns about its reach, speed—and lack of regulation on social media platforms that facilitate its impact—there has been an increasing research focus on how exposure to false or misleading content shapes belief and behavior (Zhu et al., 2012; Pennycook et al., 2018). A rapidly growing area of research in cognitive psychology has focused on the role of simple repetition in shaping people’s beliefs and impressions about information they encounter. This research shows that while increasing repetitions can lead people to be more likely to endorse a claim as true, just one exposure to content shapes how people think about a given claim—nudging people toward believing the claim is more true compared to a novel claim (Hasher et al., 1977; Dechêne et al., 2010; Hassan and Barber, 2021). This finding addresses the applied concern regarding the impact of repeated exposure of claims on belief when people are often passively consuming information as they scroll through social media feeds. Another applied concern is how the extent to which people interact with repeated information increases or decreases the magnitude of this repetition effect. We examine this question, drawing on cognitive processing perspectives, in three experiments reported here.

How does simple exposure to ideas, claims and opinions shape the lens through which we view them? The answer to this question, with robust empirical support, is that relative to information one has not seen before, repeated information is judged as more true—a phenomenon called the Illusory Truth Effect (ITE) (Hasher et al., 1977; Begg et al., 1992; Corneille et al., 2020). Mimicking repeated exposure to ideas in daily life, in typical ITE studies, participants see a series of trivia claims at an initial encoding phase. After a delay (of a few minutes, hours, or months), in a test phase, participants then see another series of claims (half they’ve seen before, and half are new). In this test phase, participants decide whether claims are true (either using forced choice responses or truth rating scales). The ITE has been widely studied and the effect holds across a wide variety of materials including trivia claims, opinions, health-related statements and fake news headlines (Arkes et al., 1991; Dechêne et al., 2010; Pennycook et al., 2018, 2020; Freeze et al., 2021; Pillai and Fazio, 2021; Unkelbach and Speckmann, 2021). The ITE is present even when one is put in a context where they could otherwise draw on semantic or general knowledge to assess the veracity of a claim (Bacon, 1979; Dechêne et al., 2010; Fazio et al., 2015). The ITE also occurs when people are presented with claims that are clearly low in plausibility. For example, one study found that even when people view implausible claims such as, “A sari is a short, pleated skirt worn by men in Scotland,” simple repetition increases their perceived truth (Fazio et al., 2019; Lacassagne et al., 2022). Moreover, warning people about the ITE or of the presence of false information as they review the claims reduces the effect, but does not eliminate the role of repetition in judgments of truth (Nadarevic and Aßfalg, 2017; Calio et al., 2020; Calvillo and Smelter, 2020; Jalbert et al., 2020).

How does repetition exert these effects on judgment? An account of the ITE that has amassed broad empirical support is that the illusory truth effect emerges as a result of processing fluency (e.g., Reber and Schwarz, 1999). According to this body of research, repetition enhances the ease of processing statements and people draw on this metacognitive cue in evaluating truth. That is, people notice the ease or difficulty in processing information in their day-to-day experience and draw on this metacognitive cue to inform their truth judgments (Reber and Schwarz, 1999; Unkelbach, 2007). In the context of the ITE, repetition is the source of variation in processing fluency. Unkelbach and Rom (2017) further developed this theoretical account in the referential theory of the ITE which assumes that the experience of processing fluency is driven by initial activation and then reactivation of semantic references in conceptual knowledge networks. People use the boost in fluency or relatively larger conceptual reactivation experienced with repeated statements as an inferential cue to truth. In short, the referential theory of the ITE emphasizes the role of conceptual fluency in driving the ITE. Evidence for this account of the ITE has emerged from studies varying the semantic processing of claims. For example, compared to when people simply read claims, when people consider and describe how the claims refer to them personally, this more elaborate encoding activity leads to a larger ITE (Unkelbach and Rom, 2017). Other research has also shown that those who report having a more elaborate thinking style—a stronger Need for Cognition—and who may elaborate more on statements they encounter can also demonstrate a larger ITE (Newman et al., 2020, cf. Boehm, 1994; De keersmaecker et al., 2020). Other findings using different manipulations of semantic elaboration align. For instance, when people see more coherent semantic references at encoding such as verbatim repetition of claims, rather than more limited topic repetition, the ITE is larger (e.g., verbatim repetition: “A hen’s body temperature is about 104 degrees Fahrenheit”; topic repetition: “Hen’s body temperature”; Begg et al., 1985).

### 1.1 Secondary tasks in the ITE paradigm

In everyday processing of information, one can be engaged in focused and elaborate processing. However, given our increasingly saturated information environments, we may be engaged in more surface level processing, especially in online contexts (Boczkowski et al., 2017, see also Newman et al., 2022). Under these conditions, where cognitive resources are more distributed, the referential theory might lead us to expect a relatively smaller ITE compared to when people are focused solely on encoding a given claim. The reason for this smaller ITE is due to the reduced opportunity for semantic elaboration under these conditions (see Lozito and Mulligan, 2006; Knott and Dewhurst, 2007). To date, very little ITE research has considered the impact of distracting participants from encoding the semantic elements of the claims. While we know that semantic oriented goals can increase the magnitude of the ITE, it is possible that other goals that draw people’s attention to surface-level characteristics of content they encounter may somewhat limit semantic encoding—and referential activation—thus reducing the magnitude of the ITE.

Another possibility is that beyond a referential account of the ITE, in adding a secondary task or distracting people, we might be simply directing attention away from thinking about truth. When people focus on detecting truth at encoding, they show a smaller ITE (e.g., Brashier et al., 2020; Jalbert et al., 2020). Given the lack of research on the impact of secondary tasks, we initially expected that a secondary task may distract people in this manner by directing goal orientation away from truth and thus perhaps producing a larger ITE. This was our pre-registered hypothesis.

Borrowing an adapted ITE paradigm from another line of research (Ly et al., 2023), we examine the question about the impacts of a secondary task in a series of three experiments. Our goal was to understand how asking participants to attend to linguistic level features of a claim (drawing their attention to less semantic-based aspects of the claim) may influence their susceptibility to the ITE. In Ly et al. (2023) (*N* = 1,102), we examined the ITE under different processing conditions and used a paradigm inspired by the Transfer-Appropriate Processing (TAP) framework (see Morris et al., 1977; Graf and Ryan, 1990; Meier and Graf, 2000). In drawing on the TAP framework, we added an ongoing linguistic-based secondary task to the classic ITE paradigm that required participants to attend to linguistic features of each claim (e.g., count the vowels in the underlined word). In our original research we were interested in whether giving people a matching or mismatching secondary task influenced the magnitude of the ITE. In the current study, we use this secondary task to investigate how the impact of a distraction task—with a linguistic focus—influences the magnitude of the ITE compared to a no secondary task baseline control.

Adding a linguistic-based secondary task affords two opportunities. First, we can examine how adding a distraction task impacts the magnitude of the ITE compared to a control condition, where participants are simply reading. Second, because the answers to this task can be coded for accuracy, we can also examine how performance on this task influences the magnitude of the ITE. We were able to explore the answer to this second question by examining data that we already had from Ly et al. (2023). We first coded people’s responses to the linguistic task (e.g., counting vowels in the underlined word of each claim) to calculate participants’ accuracy. We then calculated the magnitude of the ITE for each participant. In these exploratory analyses of accuracy scores, a systematic finding emerged between secondary task accuracy and the magnitude of the ITE. We found that higher accuracy correlated with a larger ITE, a kind of individual difference metric capturing variation in task performance. This analysis was not included in Ly et al. (2023), because (1) there was no control condition where participants simply read claims without a secondary task and (2) these analyses were not pre-registered.^1^ Nonetheless, these exploratory findings led to the current study in which our key focus was to compare the magnitude of the ITE when there is a secondary task to a control condition where no tasks are involved. Secondly, because the answers to this task can be coded for accuracy, we can examine how performance on this task influences the magnitude of the ITE.

In the ITE literature, there is to our knowledge no work that has comprehensively explored the effect of and accuracy of a secondary task as a way to capture the impact of dual processing requirements on the ITE. Existing studies have had participants make ratings at encoding, (e.g., rating interest levels or making semantic categorizations at encoding; Ladowsky-Brooks, 2010; Nadarevic and Erdfelder, 2014; Nadarevic and Aßfalg, 2017; Nadarevic et al., 2018). While we may consider these ratings as secondary tasks, these ratings encourage participants to focus on the entire claim. These tasks to not, however, interrupt reading or reduce semantic encoding of the claim. Indeed, it may be the case that in the current experiments we can index the extent to which the dual tasks impact some participants more than others (individual resilience to dual tasks) and the consequence for ITE. In everyday life, people are often consuming information and multi-tasking (Boczkowski et al., 2017; Newman et al., 2017). It is, therefore, important to consider how the ability to dual task can impact the effect of repetition on perceived truth. Further, this is an important question to address when considering the referential theory of the ITE which emphasizes the activation of semantic networks as a core underlying mechanism of the ITE. What happens when people may be under cognitive demands that could draw their attention away from deeper semantic processing to more surface level features of the content they encounter? These are important theoretical and applied questions when one considers the variation in engagement with content or the task at hand on different online or social media platforms. Our current study addresses one instantiation of “ongoing cognitive processing” when consuming information online and provides early evidence into how factors such as attentiveness or cognitive capacity may shift the impact of repetition on peoples’ truth perception.

### 1.2 Current study

To be transparent about the genesis of this line of research, we mentioned earlier that we initially found a positive relationship between participants’ task accuracy and the magnitude of the ITE in the exploratory analyses of another ITE project (Ly et al., 2023). In the current study, across three experiments, we further examined this curious finding. We followed the general method of the Ly et al. (2023) paper but with a few key changes. A key difference in our three experiments presented here is that we included a control condition where participants completed no tasks at encoding or test. By doing so, we can contextualize how the presence of a secondary task itself alters the magnitude of the ITE. We refer to the control condition as the No Task condition throughout. These experiments extend the current understanding of the referential theory of the ITE by testing how tuning people’s processing to focus on a specific feature of a claim (e.g., counting vowels in one of the words) can moderate the ITE. Further, we examined how participants’ attentiveness to a secondary task (as indexed by accuracy scores) may moderate the ITE. All our experiments were pre-registered which is available along with Supplementary material and datasets, via the following link: https://osf.io/khp45/?view_only=5cde30c1841c43d1a1d9f0c33b01b963.

## 2 Experiment 1

Prior data has shown that the ITE is associated with people’s performance on a secondary task (Ly et al., 2023; unreported data). In Experiment 1, we aimed to replicate the exploratory findings of the project discussed earlier and to understand the extent to which ongoing secondary tasks impact the ITE. Firstly, how does the presence of an ongoing secondary task moderate the magnitude of the ITE? To address this question, we included a No Task (control) condition, allowing us to contextualize the impact of the ongoing secondary tasks. Secondly, to what extent does participants’ performance on the secondary task impact the ITE? We initially hypothesized that the ITE may be larger where a secondary task condition functioned to distract people from the task of considering truth (reducing skepticism; see Jalbert et al., 2020), but as detailed throughout we find accumulating support for the opposite. When experiments are considered in concert, a secondary task tends to reduce the magnitude of the ITE. Lastly, in replicating the initial accuracy finding, we expect there to be a positive correlation between the size of the ITE and participants’ overall accuracy scores on the secondary task.

### 2.1 Experiment 1 method

#### 2.1.1 Participants

The meta-analysis by Dechêne et al. (2010) shows that a sample size of 54 would be required to detect an ITE (*d* = 0.50,95%*CI*[0.43,0.57]) in a repeated measures design. However, we used a method based on Jalbert et al. (2020) where participants receive no alert at encoding that some statements may be false, leading to a larger estimated effect size of (*d* = 1.42,95%*CI*[1.18,1.67]). Based on our G*Power calculations (Faul et al., 2007), accounting for the lower estimates of the ITE such as an *f* = 0.20, applying *a* = 0.05 and power (1-β) = 0.95, the estimated *N* was at least 100. Considering the likelihood of exclusions, and with the goal of gaining sufficient power, we aimed to obtain 150 participants online through Amazon Mechanical Turk (MTurk). A total of 149 participants participated in the study. After exclusions, the total sample size was *N* = 132. In accordance with the exclusion criteria described in the pre-registration, we excluded 16 participants for checking the answers online and 1 participant for failing to complete the study. A sensitivity analysis for Experiment 1, setting the α level at 0.05 and the sample size at 132, revealed we had 95% power to detect an effect size (*f*) of 0.17. The total duration of the experiment was approximately 25 min. Participants (including those who were excluded) were compensated 3.00USD.

#### 2.1.2 Design

We used a 2 (repetition: repeated, new) × 3 (secondary task: vowel-counting task, shoebox task, no task) mixed design, manipulating repetition within-subjects and secondary task condition between-subjects.^2^

#### 2.1.3 Materials

##### 2.1.3.1 Trivia claims

The trivia claims covered different topics such as sports, animals, food and geography. We selected the trivia claims from a larger set of normed general knowledge claims (Jalbert et al., 2020; see Supplementary Tables 1A, B: see text footnote 1). Half the claims were true and half were false. We counterbalanced the presentation of the trivia claims so that all claims appeared equally often as repeated or new at test. We used the trivia claims in all the experiments in the current study.

##### 2.1.3.2 Secondary task

Like in Ly et al. (2023), we borrowed two different tasks from a TAP study (Franks et al., 2000) which focused participants’ processing on more tangential linguistic (vowel-counting) or semantic (size estimation) features of a claim. For the *vowel-counting task*, we had participants respond yes or no to the question, “Does the underlined word have fewer than 3 vowels?” For the *shoebox task*, we asked participants to answer yes or no to the question, “Is the object/subject (underlined word) in the statement bigger than a shoebox?” We fully randomized whether participants would complete the vowel-counting or shoebox task.

#### 2.1.4 Procedure

##### 2.1.4.1 Encoding phase

**FIGURE 1:**
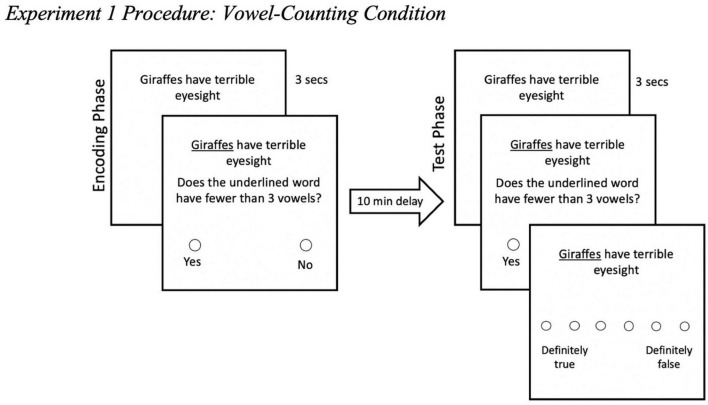
Experiment 1 procedure: vowel-counting condition. Participants completed either the vowel-counting task (shown here) or the shoebox task (not shown). This example captures what participants saw in the vowel-counting task. In an encoding-test mismatch condition, participants would complete a different task at test from what they did at encoding (in this case, the shoebox task at test).

**FIGURE 2:**
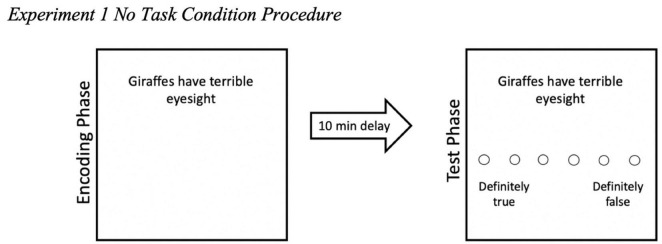
Experiment 1 no task condition procedure. This example captures what participants in the no task condition saw—no tasks and no initial 3-s exposure to the trivia claims.

We used Qualtrics to develop and present the materials online. This study was conducted online and thus, informed consent was obtained through participants clicking to indicate that they had read the Participant Information Sheet and consented to participating in the study. We want to note that we obtained informed consent in the same manner throughout all the Experiments reported here. In the initial encoding phase, we set it up to mimic a typical ITE paradigm with one key change—participants completed a secondary task when they were presented with a trivia claim. Participants were randomly assigned to one of three secondary task conditions: vowel-counting task, shoebox task, or the no task condition. In all three conditions, participants saw a total of 18 trivia claims at encoding. Each trivia claim was presented individually in black font (size 14pt) against a plain white background. In the vowel-counting and shoebox task conditions, for all 18 trials of the encoding phase, participants saw the trivia claim for 3 seconds and then an underline appeared under a target word. Then the tangential task appeared for participants to complete (see Figure 1). After completing the task, participants proceeded to the next trivia claim (see Supplementary Table 3 for encoding phase instructions). We included the initial 3-s exposure to each trivia claim prior to the task and underlined target word appearing to ensure participants read the entire trivia claim instead of focusing only on the underlined words to complete the task at every trial. For the *no task* condition, it was the standard ITE method which served as a control. For participants in the *no task* condition, at encoding, participants simply had to read each trivia claim as it appeared (see Figure 2). A total of 18 trivia claims were automatically presented over a 3-min duration. Each claim appeared for 10 s. The 3-min duration was similar to the duration for the self-paced vowel-counting and shoebox task conditions.

##### 2.1.4.2 Delay phase

Following the encoding phase, there was a delay phase where we asked participants to read passages and answer comprehension questions. Each passage covered a topic that was unrelated to the tangential tasks or trivia claims. The delay phase lasted 10 min.

##### 2.1.4.3 Test phase

After the delay phase, participants completed the test phase where they saw another series of claims (36 in total; presented in randomized order) and were told that half the claims had appeared in the encoding phase (18) and half were new (18). Similar to the encoding phase, in the test phase participants were given a secondary task to complete for each trivia claim they saw. Those who were randomly assigned to the vowel-counting task condition completed the same task that they did at encoding. Participants who were randomly assigned to the shoebox task condition completed the same task as they did at encoding. A key difference from the encoding phase was that after responding to the secondary task, we asked participants to make truth ratings about how true or false each trivia claim was. Participants made truth ratings by using a six-point Likert Scale ranging from “Definitely True” (1) to “Definitely False” (6). After completing the test phase, participants answered demographic questions (e.g., gender, age, political orientation and religiosity) (see Supplementary Table 4 for the test phase instructions). For those in the no task condition, participants simply read the claims that appeared to them and then made truth ratings using the same Likert-type scale described above.

##### 2.1.4.4 Data quality checks

We used several data quality checks to ensure that the data collected from MTurk were high quality. Firstly, following solutions proposed by Hauser et al. (2018), to ensure an attentive sample from MTurk, we only collected data from MTurkers based on their past data quality (e.g., Human Intelligence Task (HIT) Approval Ratio). Only MTurkers with a ≥ 95% HIT Approval Rate were able to complete our study as they have been found to score higher on measures of attentiveness (Peer et al., 2014). We also included a Captcha feature at the end of the study to screen for bots. Secondly, to improve data quality, we included our own “BotCheck” question toward the end of the study to screen for bots. We asked participants to type out a trivia claim that they found interesting. Those who failed to give a comprehensive answer (e.g., random unrelated phrases or strings of words/numbers) were assumed to be bots and excluded from analysis. Applying these exclusion criteria, as noted above, we excluded 16 people.

### 2.2 Experiment 1 results

Recall that in Experiment 1, we aimed to investigate two questions. Firstly, does including a secondary task impact the magnitude of the ITE? The answer is no. We found an ITE across secondary task conditions; however, there was no difference in the size of the ITE between conditions with tasks compared to no tasks. Secondly, does participants’ accuracy on the secondary task correlate with the magnitude of the ITE? The answer is yes. We found that there was a positive association between the size of the ITE and overall task accuracy—the ITE magnitude increased with increasing accuracy on the secondary item-by-item task.^3^

#### 2.2.1 Was there an ITE?

**FIGURE 3:**
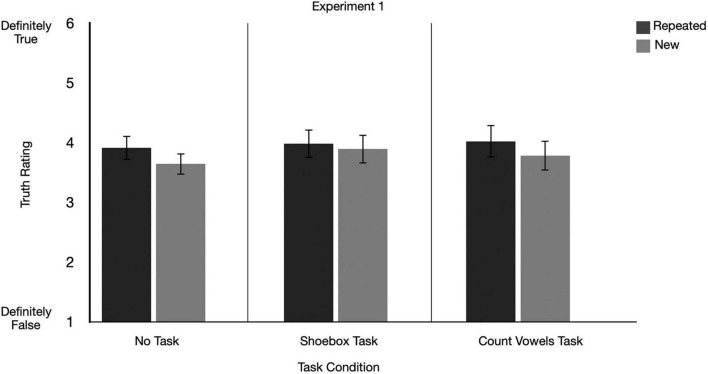
Graphs depict mean truth ratings for repeated and new claims across secondary task conditions for Experiment 1. For Experiment 1, in the shoebox task and count vowel task condition, participants completed tasks at encoding and at test. Error bars represent 95% confidence intervals.

We ran a 2 (repetition: repeated, new) × 3 (secondary task: vowel-counting task, shoebox task, no task) repeated measures ANOVA on the mean truth ratings. There was a significant main effect of repetition on truth ratings, indicating an ITE was present, *F*(1, 129) = 10.24, *p* = 0.002, *partial eta squared* = 0.07, 90% *CI* [0.02, 0.15]. There was no significant main effect of secondary task condition, *F*(2, 129) = 0.61, *p* = 0.543, *partial eta squared* = 0.01, 90% *CI* [0.00, 0.04] (see Figure 3).

#### 2.2.2 Did the size of the ITE differ depending on whether participants were given tasks?

There was no significant interaction between the ITE and secondary task condition, *F*(2, 129) = 1.54, *p* = 0.219, *partial eta squared* = 0.02, 90% *CI* [0.00, 0.07]. Thus, there was no significant difference in the size of the ITE between Vowel-counting Task, Shoebox Task or No Task conditions (see Figure 3).

#### 2.2.3 Was the magnitude of the ITE associated with participants’ task accuracy?

**FIGURE 4:**
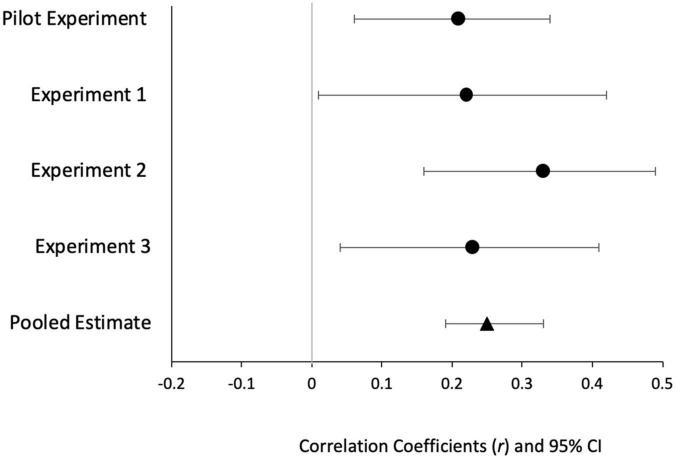
Mini-meta analysis of effect size (*r*) for the relationship between overall task accuracy and ITE. Effect sizes (*r*) for the relationship between overall task accuracy and the ITE across all experiments. The pooled estimated mean was obtained following a fixed effects mini meta-analysis in which experiments were weighted by sample size (*N*). These effects represent 95% confidence intervals.

We found a small but significant correlation between truth difference and overall accuracy, *r*(87) = 0.22, *p* = 0.040, 95% *CI* [0.01, 0.42] (see Figure 4). This finding demonstrates that as participants’ accuracy on the secondary task increased, so did the size of the ITE. This effect remained after we removed outliers (those with 100% and <50% scores), *r*(83) = 0.23, *p* = 0.040, 95% *CI* [0.02, 0.43].

### 2.3 Experiment 1 discussion

In Experiment 1, we aimed to investigate how including an ongoing secondary task affects the magnitude of the ITE. We found no differences in the size of the ITE depending on whether a task was present (No Task vs. a Task condition) or the type of task participants completed (shoebox or vowel-counting task). This finding suggests that the ITE may not be impacted when participants are engaged in dual-tasking throughout the experiment. But this experiment considered the impact of a secondary ongoing task which occurred at both encoding and retrieval. It is possible that temporal placement of the secondary task matters. Indeed, evidence suggests that processing shifts at encoding tend to influence the size of the ITE, rather than activities at test (Unkelbach, 2007; Garcia-Marques et al., 2016; Brashier et al., 2020; Jalbert et al., 2020; Newman et al., 2020). Specifically, evidence shows that variables at encoding—that place less emphasis on veracity of claims at encoding—tend to increase the size of the ITE (Unkelbach, 2007; Jalbert et al., 2020; Newman et al., 2020). Hence, replicating Experiment 1, in Experiment 2 we examined whether the temporal placement of our ongoing secondary task (at encoding or at test) influenced the general magnitude of the ITE.

**FIGURE 5:**
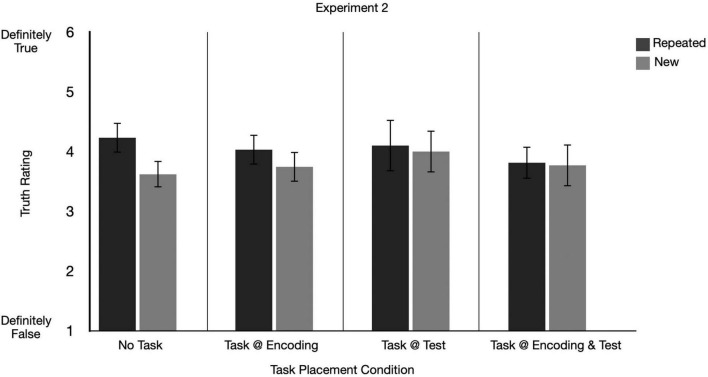
Graphs depict mean truth ratings for repeated and new claims across secondary task conditions for Experiment 2. Error bars represent 95% confidence intervals.

**FIGURE 6:**
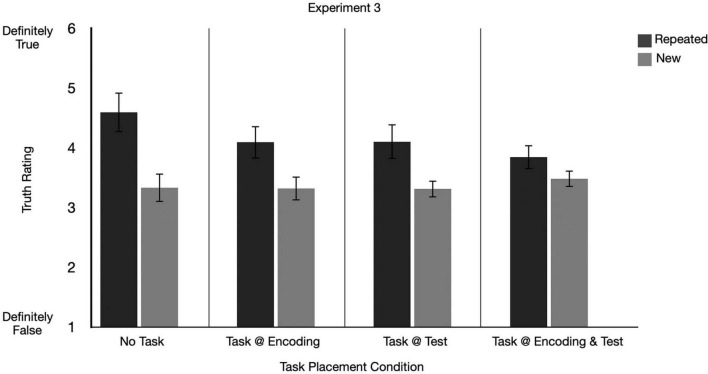
Graphs depict mean truth ratings for repeated and new claims across secondary task conditions for Experiment 3. Error bars represent 95% confidence intervals.

We want to note that the duration of the encoding period of the No Task and Task conditions varied (e.g., with No Task lasting 10 s as claims are automatically presented and Task conditions having the initial 3s reading time prior to completing a task). Our data in the Task at Test only condition of Experiment 2 and 3 address this concern. In this Task at Test condition, participants experience the same exposure conditions (duration) as those in the control condition. Despite the matched encoding conditions, Figures 5, 6 show that the ITE in the No Task condition is always larger than the ITE in the Task at Test condition—suggesting that differences in the exposure duration do not lead to changes in the overall pattern.

Further, in line with the exploratory analysis of Ly et al. (2023)’s data, we found that as participants’ accuracy on the ongoing secondary tasks increased, so did their ITE. Assuming that performance on the tasks indicates how engaged or attentive participants were when completing tasks, these findings suggest that increased attentiveness to the secondary task may increase people’s susceptibility to the ITE. Another interpretation of this pattern is that it indexes people’s ability to dual task in this context (Kane et al., 2001; Engle, 2002, 2018). We consider these accounts further in the General Discussion. In Experiment 2, we further attempt to understand and replicate this correlational finding and manipulate the placement of the secondary ongoing task.

## 3 Experiment 2: secondary task reduces the ITE and higher accuracy correlates with ITE

In Experiment 2, we aimed to replicate our initial findings regarding a secondary task, but further also investigate the impact of *when* participants completed the ongoing secondary tasks: at the initial encoding phase, test phase or both. We also aimed to examine whether the accuracy pattern held. We changed our design such that we had only one kind of secondary task condition—the linguistic vowel counting task, which most closely aligned with the goal of examining the impact of distracting people to surface-level features of a claim.

### 3.1 Experiment 2 method

#### 3.1.1 Participants

Due to an additional between-subjects condition, we posted 200 participant slots on MTurk. The final sample size was *N=181*. We excluded one participant for failing to complete the study and 18 participants for failing the BotCheck question. A sensitivity analysis for Experiment 2, setting the α level at 0.05 and the sample size at 181, revealed we had 95% power to detect an effect size (*f*) of 0.16. The total duration of the experiment was approximately 25 min and participants (including those excluded) were compensated 3.00USD.

#### 3.1.2 Design

We used a 2 (repetition: repeated, new) × 4 (secondary task placement: task at encoding, task at test, task at encoding *and* test, no task) mixed design, manipulating repetition within-subjects and secondary task placement between-subjects.

#### 3.1.3 Materials and procedure

Experiment 2 used the same materials and followed the same procedure as Experiment 1, but with the following changes. Firstly, we removed the shoebox task from the design and only had participants in this experiment complete the vowel-counting task. We chose to only use the vowel-counting task because the task does not require semantic processing and simplifies coding for accuracy.^4^ Secondly, we included additional between-subject conditions such that participants were randomly assigned to one of four secondary task placement conditions:(1) task at encoding, (2) task at test, (3) task at encoding and test, or (4) no task condition. In the *task at encoding* condition, participants completed the vowel-counting task at the encoding phase only. In the *task at test* condition, participants completed the vowel-counting task only at the test phase. In the *task at encoding and test* condition, participants completed the vowel-counting task at both phases. For the *no task* condition, we used the same procedure as the no task condition in Experiment 1.

### 3.2 Experiment 2 results

In Experiment 2, we wanted to know whether the magnitude of the ITE changed depending on when participants were given the secondary task. Participants either completed the vowel-counting task at encoding, at test, or at encoding and at test, or were not given a task at all. We found a difference in the size of the ITE between the secondary task placement conditions. The ITE was largest in the *no task* condition compared to the rest of the secondary task conditions. Notably, although Experiment 1 also had a *no task* condition, this pattern of results only emerged in Experiment 2 (and 3). Secondly, like in the previous experiments, we found a positive correlation between task accuracy and the size of the ITE.^5^

#### 3.2.1 Was there an ITE?

We ran a 2 (repetition: repeated, new) × 4 (secondary task placement condition: task at encoding, task at test, task at encoding and test, no task) repeated measures ANOVA on the mean truth ratings. There was a significant main effect of repetition on truth ratings, indicating an ITE was present, *F*(1, 177) = 25.37, *p* < 0.001, *partial eta squared* = 0.13, 90% *CI* [0.06, 0.20]. There was no significant main effect of secondary task placement condition, *F*(3, 177) = 0.73, *p* = 0.537, *partial eta squared* = 0.01, 90% *CI* [0.00, 0.04] (see Figure 5).

#### 3.2.2 Did the size of the ITE differ depending on when participants completed tasks?

There was a significant interaction between repetition and secondary task placement condition, *F*(3, 177) = 2.98, *p* = 0.033, *partial eta squared* = 0.05, 90% *CI* [0.00, 0.10]. This result shows that the size of the ITE differs across secondary task placement conditions: *task at encoding*, *task at test, task at encoding and test* and *no task* (see Figure 5). Indeed, in a follow-up paired samples *t*-test on the difference between mean truth ratings for repeated and new items, by secondary task placement condition, we found a significant ITE in the *no task*, *task at encoding* and *task at test* condition, but unlike Experiment 1, no ITE in the *task at encoding and test* condition. Notably as evidenced by the raw mean differences within each condition and associated confidence intervals, the magnitude of the ITE was largest in the *no task* condition, (*t*(45) = 3.97, *p* < 0.001, *raw mean difference* = 0.61, 95%*CI*[0.30,0.91]*M*_*Repeated*_ = 4.23, *SD* = 0.89; *M*_*New*_ = 3.62, *SD* = 0.72). There was a significant, but smaller, ITE in the *task at encoding* condition, (*t*(43) = 2.14, *p* = 0.038, *raw mean difference* = 0.29, 95%*CI*[0.02,0.57]*M*_*Repeated*_ = 4.03, *SD* = 0.89; *M*_*New*_ = 3.74, *SD* = 0.83), and the *task at test* condition, (*t*(50) = 2.99, *p* = 0.004, *raw mean difference* = 0.41, 95%*CI*[0.13,0.69]*M*_*Repeated*_ = 4.19, *SD* = 1.05; *M*_*New*_ = 3.80, *SD* = 0.99). There was no significant ITE in the *task at encoding and test* condition, (*t*(39) = 0.47, *p* = 0.639, *raw mean difference* = 0.04, 95% *CI* [0.13, 0.21], *M*_*Repeated*_ = 3.78, SD = 0.90; *M*_*New*_ = 3.74, SD = 0.92). In our three experiments, this was the only time that we failed to observe the ITE in this condition. Although these results support the general prediction that the temporal placement of the task may impact the size of the ITE, it was not in the direction we expected. We further examine the extent to which these findings replicate in Experiment 3.

#### 3.2.3 Was the magnitude of the ITE associated with participants’ task accuracy?

A correlational analysis revealed a significant positive correlation between truth difference and overall accuracy (%), *r*(114) = 0.33, *p* < 0.001; 95%*CI*[0.16,0.49] (see Figure 4). As in Experiment 1 [and in Ly et al. (2023)], these results show that as participants’ accuracy on the secondary task increases (higher attentiveness paid to the secondary task), the ITE increases. After removing the outliers (those with 100% and >50% accuracy scores), we still found a significant correlation between truth difference and overall task accuracy, *r*(66) = 0.44, *p* < 0.001; 95%*CI*[0.23,0.62].

### 3.3 Experiment 2 discussion

One key goal of Experiment 2 was to investigate the extent to which the size of the ITE depends on when participants completed the item-by-item secondary tasks throughout the experiment. We found that the size of the ITE was largest when participants did not complete any secondary task (in the *no task* condition) compared to the other secondary task placement conditions. Although we did not find an effect of the secondary task in Experiment 1, this finding suggests that a secondary task may change the nature of the ITE itself.

Secondly, we expected but did not find that compared to when the task was given at test or at both phases, having the *task at encoding* would lead to a larger ITE. In Experiment 2, we predicted and found that higher accuracy was significantly correlated with a larger ITE—notably a third replication of this finding in this line of research (see Figure 4).

As this is the first time we found a significant interaction between claim repetition status and the secondary task placement condition, we ran a replication of Experiment 2 using a university participant pool sample.

## 4 Experiment 3: replicating Experiments 1 and 2 with university participants

In Experiment 3, we aimed to replicate Experiment 2 with a different participant pool. For Experiment 3, instead of collecting data via MTurk, we collected a university participant sample using SONA participants.

### 4.1 Experiment 3 methods

#### 4.1.1 Participants

Replicating Experiment 2, we posted 200 participant slots on SONA. Our final sample size was *N* = 162. We excluded 33 participants for failing to complete the full study and 5 were excluded for checking answers. A sensitivity analysis for Experiment 3, setting the α level at 0.05 and the sample size at 162, revealed we had 95% power to detect an effect size (*f*) of 0.16. The total duration of the experiment was approximately 25 min and participants (including those excluded) were awarded course credit.

#### 4.1.2 Design

We used a 2 (repetition: repeated, new) × 4 (secondary task placement: task at encoding, task at test, task at encoding and test, no task) mixed design, manipulating repetition within-subjects and secondary task condition between-subjects.

#### 4.1.3 Materials and procedure

Experiment 3 procedure was a direct replication of Experiment 2.

### 4.2 Experiment 3 results

In Experiment 3, we aimed to replicate Experiment 2 using a university participant pool sample. Like in Experiment 2, we found that the size of the ITE was largest in the *no task* condition compared to the other secondary task placement conditions. We also found that participants’ overall accuracy on the secondary task correlated with the size of the ITE.^6^

#### 4.2.1 Was there an ITE?

We ran a 2 (repetition: repeated, new) × 4 (secondary task placement: task at encoding, task at test, task at encoding and test, no task) repeated measures ANOVA on the mean truth ratings. There was a significant main effect of repetition on truth ratings, indicating an ITE was present, *F*(1, 158) = 101.13, *p* < 0.001, *partial eta squared* = 0.39, 90% CI [0.29, 0.47]. There was no significant main effect of secondary task placement condition, *F*(3, 158) = 2.08, *p* = 0.105, *partial eta squared* = 0.04, 90% *CI* [0, 0.08] (see Figure 6).

#### 4.2.2 Did the size of the ITE differ depending on when participants completed tasks?

There was a significant interaction between repetition and secondary task placement condition, *F*(3, 158) = 5.49, *p* = 0.0013, *partial eta squared* = 0.09, 90% *CI* [0.02, 0.16] (see Figure 6). Indeed, like in Experiment 2, a follow-up paired samples t-test revealed a significant, and largest ITE in the *no task* condition, (*t*(39) = 6.40, *p* < 0.001, *raw mean difference* = 1.26, 95%*CI*[0.86,1.65]*M*_*Repeated*_ = 4.59, *SD* = 1.08; *M*_*New*_ = 3.33, *SD* = 0.74). Relative to the *no task* condition, there was a significant but smaller ITE in the *task at encoding* condition, (*t*(40) = 4.79, *p* < .001, *raw mean difference* = 0.77, 95% *CI* [0.45, 1.10], *M*_*Repeated*_ = 4.09, SD = 0.87; *M*_*New*_ = 3.34, SD = 0.10), task at encoding and test condition, (*t*(41) = 3.11, *p* = 0.003, *raw mean difference* = 0.36, 95% *CI* [0.13, 0.59], *M*_*Repeated*_ = 3.84, SD = 0.66, *M*_*New*_ = 3.48, SD = 0.68) and the task at test condition, (*t*(38) = 5.23, *p* < .001, *raw mean difference* = 0.79, 95% *CI* [0.48, 1.09], *M*_*Repeated*_ = 4.10, SD = 0.94, *M*_*New*_ = 3.31, SD = 0.48).

#### 4.2.3 Was the magnitude of the ITE associated with participants’ task accuracy?

A correlational analysis revealed a significant positive correlation between the magnitude of the ITE and overall accuracy, *r*(101) = 0.23, *p* = 0.020, 95%*CI*[0.4,0.41]. These results show that as participants’ overall accuracy on the secondary task increases, the ITE increases (see Figure 4). After removing the outliers (those with 100% and >50% accuracy), we found a significant correlation between truth difference and overall accuracy, *r*(51) = 0.31, *p* = 0.025, 95%*CI*[0.04,0.54].

### 4.3 Experiment 3 discussion

Experiment 3 was a direct replication of Experiment 2 where we aimed to investigate two questions with a university participant pool sample, (1) whether the temporal placement of the secondary task mattered and (2) whether overall task accuracy would be associated with the magnitude of the ITE. Like in Experiment 2, our university participant sample showed a significant difference in the size of the ITE depending on whether participants completed a task. Notably, the ITE was largest in the *no task* condition compared to the other secondary task conditions (*task at encoding*, *task at test*, *task at encoding and test*). Considered together, across Experiments 2 and 3, we found a systematic effect of the *no task* condition compared to secondary tasks, but no systematic pattern regarding the temporal placement of the secondary task on the size of the ITE. The findings regarding temporal placement of the task are at odds with research that has captured a greater change in the ITE when processing manipulations are employed at encoding (Jalbert et al., 2020). We discuss this finding and the larger ITE in the *no task* condition further in the section “5. General discussion.”

Lastly, as in all experiments reported here, we again found that as overall task accuracy increased, so did the size of the ITE. We performed a mini meta-analysis to further test the robustness of this pattern of results.

#### 4.3.1 A mini meta-analysis of the relationship between secondary task performance and the magnitude of the ITE

A mini meta-analysis was performed across four experiments which include the Experiments 1 to 3 from the current study and data from one experiment of Ly et al. (2023) that we mentioned above—here it is referred to as Pilot Experiment. The mini meta-analysis reported on the magnitude of the relationship between overall task accuracy and the ITE. We used fixed effects where the mean correlation effect size was weighted by the sample size of each experiment. All the correlations were transformed to Fisher’s *z* for analyses and then converted back to Pearson correlations (Goh et al., 2016). Across the four experiments, participants’ accuracy on the task was significantly positively related to the ITE. Thus, higher overall accuracy was associated with a larger ITE (*M*_*r*_ = 0.25, *p* < 0.001, 95%*CI*[0.19,0.33]).^7^

## 5 General discussion

In the three experiments reported here, beyond simply reading claims—as is typically done in the ITE paradigm—we had participants complete a simple ongoing secondary task and measured their accuracy. Our results overall suggest, firstly, there is some evidence in two out of three experiments that the presence of a secondary task itself can reduce the magnitude of the ITE and secondly, that the magnitude of the ITE increases as participants are better at the ongoing secondary task. Further interpretation of our findings and recommendations for future research are discussed below.

### 5.1 Understanding the cost of disrupted semantic processing on the ITE

In two out of our three Experiments (Experiment 2 and 3), we found a significant interaction between repetition and task condition. The findings of Experiments 2 and 3 are consistent with studies that have shown a reduced ITE when semantic processing of a claim at encoding is disrupted or constrained. For example, Begg et al. (1985) tested whether there were differences between repeating part of a sentence compared to a full sentence in moderating the ITE. At encoding, participants were shown topic phrases (topic-repetition condition, e.g., “Hen’s body temperature”) or full trivia claims (verbatim repetition condition, e.g., “The temperature of a hen’s body is about 104 degrees Fahrenheit.”). Later, participants made truth ratings on the full claim. Begg et al. (1985) found a smaller ITE in the topic-repetition condition compared to the verbatim repetition condition. This finding aligns with those reported here where participants who had the task of attending to a specific word (in the conditions with a tangential task), rather than attending to the full claim (in control)—possibly restricting semantic activation—led to a smaller ITE compared to the No Task (control) condition. We know that the extent of semantic activation (partial vs. full activation) influences the size of the ITE (Begg et al., 1985; Ly et al., 2023). The findings of Begg et al. (1985) and those in the current study further support the referential theory of the ITE (Unkelbach and Rom, 2017) which emphasizes the role that reactivation of semantic networks plays in producing the ITE when people are re-exposed to content.

While we have shown that a secondary task can reduce the magnitude of the ITE, future research might examine the degree to which a secondary task reduces the ITE based on how much it hinders semantic processing of the claim. In the current study, we drew people’s attention to linguistic features of a claim, but we may have created only minor interference in processing meaning. Other secondary tasks that create more semantic interference may reduce the magnitude of the ITE further (see semantic interference in the misinformation effect: Loftus, 2005 and Frenda et al., 2011 for a review). For instance, asking people to remember (tangential) words that they hear while encountering the trivia claims may interrupt semantic processing to a greater extent than asking them to subsequently count vowels of a target word. Indeed, in our everyday experience, listening to content and reading something else is also a dual task we engage in regularly—listening to music or podcasts while scrolling news or social media online.

### 5.2 Attentiveness, cognitive capacity and the ITE

In our exploratory analysis of the Pilot Experiment, we found a significant positive correlation between participants’ overall task accuracy scores and the size of the ITE. In all three experiments reported here, we observed this same data pattern. This finding is consistent with research that has found that manipulations that encourage deeper processing, or people who are more likely to process information on a more elaborate level, demonstrated a larger ITE (Dechêne et al., 2010; Newman et al., 2020). However, the evidence surrounding processing style and the ITE is rather mixed in the literature. For example, Garcia-Marques et al. (2016) found that inducing System 2-like processing (more analytic processing compared to System 1) reduced the ITE. In our current study, although we found no differences in the ITE depending on task type (vowel-counting vs. shoebox; replicating our existing work; Ly et al., 2023), we do find mixed results regarding the impact of a secondary task on the ITE. However, we want to note that what does emerge as a consistent pattern across experiments is that there is a correlation between secondary task performance and the magnitude of the ITE. Given that secondary task performance is a potential indicator or index of participants’ level of attentiveness, one likely account of the patterns observed in the current study is that high performing participants who are paying more attention to the task at hand are better able to engage with the content of the claims. This deeper engagement with the content of the claims may work to facilitate conceptual processing of its semantic elements, thereby ensuring adequate references are activated in the memory network—which the referential theory of the ITE suggests is key for repetition-induced fluency at later judgments.

In further examining participants’ task accuracy scores, in Experiments 1 and 2, we also found that high and low accuracy groups show a systematic difference in the size of the ITE compared to the No Task conditions. In short, when splitting participants in all the task conditions by high vs. low accuracy, those in the high accuracy group show a similar ITE to the No Task condition. This finding suggests that the correlation may be driven by the low accuracy participants who show a significantly smaller ITE when compared to the No Task condition and points to another possible account—cognitive capacity. A similar pattern is present in Experiment 3, but is less pronounced and non-significant (see further analyses, p. S13 – S16). Considering task accuracy scores as an index of secondary task processing capacity, this cognitive capacity explanation is consistent with working memory research that shows that ongoing tasks may have more of a cognitive cost to some participants than others (Engle, 2002, 2018). In general, performance of those with high working memory capacity is relatively less impacted by ongoing cognitive tasks (i.e., task switching, increased cognitive load/task difficulty; Kane and Engle, 2000; Kane et al., 2001). We found some evidence supporting this idea—those who had high accuracy on the vowel counting task had an ITE magnitude similar to those in the control condition. That is, the ongoing secondary task did not reduce the ITE relative to the No Task condition for everyone—arguably, for those who had the capacity, there was little impact (see Supplementary Table 5). Existing research on cognitive capacity/executive function shows no correlation with the magnitude of the ITE (De keersmaecker et al., 2020). However, in that research, there was no ongoing secondary task—which may be a boundary condition to which one may observe such effects. Further research could illuminate the role of cognitive capacity and the effects we have observed here. For example, further research might include external measures of participants’ processing capacity and flexibility from the working memory literature such as the Operation Span Task (OSPAN; Unsworth et al., 2005), the Wisconsin Card Sorting (WCST) (Greve, 2001), a psychological refractory period paradigm (PRP) task (Pashler, 1991; Giesbrecht et al., 2001) or a task-switching paradigm.

Another possible explanation of our findings is that the correlation between secondary task and the ITE is driven by variations of attentiveness on the task. This raises questions surrounding data quality in online experiments—a common mode of data collection in the ITE literature. Indeed, a meta-analysis by Henderson et al. (2021) revealed that around 27% of ITE studies were conducted online. The consistently significant positive correlation between accuracy and the magnitude of the ITE suggests that there is variation in participants’ performance online and that this can have an impact on the magnitude of the ITE. Considered together, it is possible that depending on the task at hand, and perhaps even levels of interest and engagement, online studies may be capturing a smaller ITE. In future research, factors such as attentiveness and cognitive capacity may be worth considering, particularly in online environments where researchers are less able to control what participants are doing whilst they are completing the study.

## 6 Conclusion

To summarize, we examined the extent to which including a secondary task may influence the magnitude of the ITE. We found some evidence that engaging in a simple secondary task like counting the number of vowels in a target word of a claim reduces the impact of repetition on perceptions of truth. Furthermore, people’s performance on the secondary task related to the magnitude of the ITE. Upon close examination we found it was those who performed poorly on the secondary task in particular who showed a smaller ITE compared to control participants. Firstly, our findings suggest a secondary task may distract or disrupt the semantic processing of a claim—a pattern of data that is consistent with the referential theory of the ITE (Unkelbach and Rom, 2017). Secondly, our findings are also early evidence suggesting the role that attentiveness and cognitive capacity may play in moderating the ITE. Further investigation in this area and novel ITE methods may be a valuable avenue for theoretical and methodological development.

## Data availability statement

The datasets presented in this study can be found in online repositories. The names of the repository/repositories and accession number(s) can be found in the article/Supplementary material.

## Ethics statement

The studies involving humans were approved by the Australian National University Human Research Ethics Committee. The studies were conducted in accordance with the local legislation and institutional requirements. The participants provided their written informed consent to participate in this study.

## Author contributions

DL: conceptualization, methodology, data analysis, writing-original draft, and writing–review and editing. DB: conceptualization, writing–review and editing, and co-supervision. EN: conceptualization, writing-original draft, writing–review and editing, and supervision. All authors contributed to the article and approved the submitted version.

## Footnotes

1
https://osf.io/dhwke/?view_only=0f9fbbd528c240d19e1ccfa52919724e

2 We wanted to replicate the exact design of Ly et al. (2023). We, therefore, included the shoebox task in Experiment 1. In the subsequent experiments, we removed this task and used only the vowel-counting task, because the responses allowed us to code for accuracy.

3 In this and the following experiments, accuracy scores can only be obtained in conditions where a task was assigned. Thus, we cannot compute accuracy in the *no task* condition.

4 Further, analyses in Ly et al. (2023) revealed no differences in the size of the ITE or the relationship between accuracy and the ITE depending on task type.

5 See p. S11 for the same analyses run without exclusions. The pattern of results does not change from that reported in the main article.

6 See p. S12 for the same analyses run without exclusions. The pattern of results does not change from that reported in the main article.

7 A random effects approach to the mini-meta analysis yielded similar results to the fixed effects approach. We followed steps provided by Goh et al. (2016) where a one sample *t*-test was run on the Fisher’s Z transformed correlation coefficients (*r*) of each experiment and the *N* is the number of studies (*N* = 4). Results showed that across our studies, overall accuracy was significantly and positively related to the ITE (*Mr* = 0.25, 95% *CI* [0.19,0.33], *f*(4) = 10.71, *p* < 0.0001).
