# Supplementary material for: An ongoing secondary task can reduce the illusory truth effect
Source: Front Psychol. 2024 Jan 3;14:1215432. doi: 10.3389/fpsyg.2023.1215432 (PMC10792064; doi:10.3389/fpsyg.2023.1215432)
Supplement: Supplementary file 1 [file Data_Sheet_1.pdf]

Supplementary Material

An Ongoing Secondary Task Can Reduce the Illusory Truth Effect

Deva P. Ly, Daniel M. Bernstein, Eryn J. Newman*

*** Correspondence:** Dr. Eryn J. Newman: eryn.newman@anu.edu.au

1 Supplementary Table S1a

Trivia Claims Counterbalance 1 (table continues onto the following page)

Claim number	Category	Claim	True or False	Proportion of true responses	Count Vowel Answer	Shoebox Answer
CB1.1	Sports	Volleyball was originally called mintonette	True	0.35	No	Yes
CB1.2	Animals	The flamingo's pink color comes from carotenoid pigments in its food	True	0.56	No	Yes
CB1.3	Geography	Canada is the second largest country in the world in area	True	0.61	No	Yes
CB1.4	Geography	The largest European glacier is Vatnajökull on Iceland	True	0.54	No	Yes
CB1.5	Science	In almost all human populations of newborns, there is a slight excess of males	True	0.46	Yes	Yes

CB1.6	Animals	Domesticated goats are descended from the pasang	True	0.49	Yes	Yes
CB1.7	Food	Most limes have more acid than lemon	True	0.63	Yes	No
CB1.8	Geography	Lake Baikal is the world's largest freshwater lake by volume	True	0.52	Yes	Yes
CB1.9	Animals	The mouth of a sea urchin is on its top	False	0.58	Yes	No
CB1.10	Food	The grape plant is a large herb	False	0.35	Yes	No
CB1.11	Food	Dough is boiled in the process of making croissants	False	0.52	Yes	No
CB1.12	Science	Neptune is part of the Kuiper belt	False	0.47	No	Yes
CB1.13	Sports	The longbow was invented after the crossbow	False	0.39	Yes	No
CB1.14	Animals	Sheep are a type of tylopod mammal	False	0.44	Yes	Yes
CB1.15	Geography	The Caspian Sea is the lowest body of water on the surface of the Earth	False	0.63	Yes	Yes
CB1.16	Geography	The Nile river flows southward	False	0.51	Yes	Yes

CB1.17	Sports	Bike riding is the first event in a triathlon	False	0.56	Yes	Yes
CB1.18	Science	The liquid metal inside a thermometer is magnesium	False	0.54	No	No

Note. The claims have been normed and the total average proportion of people responding "true" to all 18 items is 0.51.

2 Supplementary Table S1b

Trivia Claims Counterbalance 2 (table continues onto the following page)

Claim number	Category	Claim	True or False	Proportion of true responses	Count Vowel Answer	Shoebox Answer
CB2.1	Animals	Moose may dive underwater while feeding	True	0.39	No	Yes
CB2.2	Food	The Colchester is a popular type of oyster	True	0.41	Yes	No
CB2.3	Geography	Taboga Island is in Panama	True	0.46	Yes	No
CB2.4	Science	Xylem is the water-transporting tissue in plants	True	0.56	Yes	No
CB2.5	Sports	Dart boards are commonly made of sisal	True	0.57	Yes	No
CB2.6	Animals	Snakes lack moveable eyelids	True	0.58	Yes	Yes
CB2.7	Food	Cabbages are in the mustard family	True	0.54	No	No
CB2.8	Science	The sun constitutes more than 99 percent of the entire mass of the solar system	True	0.60	Yes	Yes

CB2.9	Animals	The otter belongs to the squirrel family	True	0.43	Yes	No
CB2.10	Food	The corn was first domesticated by native peoples in Argentina	False	0.42	Yes	No
CB2.11	Geography	The monetary unit in Afghanistan is the rupee	False	0.48	No	No
CB2.12	Food	Spain produces most of the world's almonds	False	0.50	Yes	Yes
CB2.13	Sports	The sport involving a snowboard is believed to have originated in Europe	False	0.53	No	Yes
CB2.14	Geography	The highest waterfall in the world is in Argentina	False	0.54	No	Yes
CB2.15	Sports	Candlepins is the most widely played variation of bowling	False	0.63	No	Yes
CB2.16	Science	In chemistry, a mass spectrometer is used to separate substances into its constituent parts according to color	False	0.63	No	Yes
CB2.17	Geography	The Carpathian Mountains form a high wall between France and Spain	False	0.52	No	Yes
CB2.18	Animals	Giraffes have terrible eyesight	False	0.51	No	Yes

Note. The claims have been normed and the total average proportion of people responding "true" to all 18 items is 0.52.

3 Supplementary Table S3

Encoding Phase Instructions Used Across the Pilot Experiment, Experiments 1, 2 and 3

Encoding Phase Instructions by Condition
For the Vowel-counting task and Shoebox task conditions:<br><br>"For approximately the next three minutes, you will see a series of trivia statements.<br><br>Your task will be to read the trivia statements carefully as they are presented and then answer the question that appears below each trivia claim.<br><br>When you are ready to begin, press the next button below."
For the No Task condition:<br><br>"For approximately the next three minutes, you will see a series of trivia statements.<br><br>The trivia statements will be presented automatically - there is no need to press any buttons. Please read the trivia statements carefully as they are presented, but do not do anything else.<br><br>Press the next button to begin."

4 Supplementary Table S4

Test Phase Instructions Used Across the Pilot Experiment, Experiments 1, 2 and 3

Test Phase Instructions by Condition
For the Vowel-counting Task condition:<br><br>"You will now see another series of trivia statements appear on the screen. Half of the statements you have already seen and half are new.<br><br>First, you will be asked to **count the number of vowels** that is present in the underlined word.<br><br>Next, you will be asked to assess whether each claim is true or false. Please read the statement carefully and answer the following question:<br><br>**Is this statement true or false?**<br><br>You will be asked to answer this question on a scale from definitely true to definitely false."<br><br>"It is important that you respond as quickly as possible, but not so quickly that you start making errors."<br><br>"Please do not search the answers online while you are completing the study; if you are unsure of an answer, please just make your best guess."<br><br>"Now you will see a series of trivia statements.<br><br>As a reminder, for each statement you will be **counting the number of vowels** in the underlined word and then assess whether the statement is true or false.<br><br>Please go on to the next page to begin."
For the Shoebox Task condition:<br><br>"You will now see another series of trivia statements appear on the screen. Half of the statements you have already seen and half are new.<br><br>First, you will be asked to **state whether the subject/object in the statement is bigger than a shoebox**.<br><br>Next, you will be asked to assess whether each claim is true or false. Please read the statement carefully and answer the following question:

Is this statement true or false?

You will be asked to answer this question on a scale from definitely true to definitely false."

"It is important that you respond as quickly as possible, but not so quickly that you start making errors."

"Please do not search the answers online while you are completing the study; if you are unsure of an answer, please just make your best guess."

"Now you will see a series of trivia statements.

As a reminder, for each statement you will **state whether the subject/object in the statement is bigger than a shoebox** and then assess whether the statement is true or false.

Please go on to the next page to begin."

For the No Task (control) condition:

"You will now see another series of trivia statements appear on the screen. Half of these statements are ones that you have already seen, and half are new.

You will be asked to assess whether each claim is true or false. When you see each statement appear on the screen, please read it carefully and answer the following question:

Is this statement true or false?

You will be asked to answer this question on a scale from definitely true to definitely false."

"It is important that you respond as quickly as possible, but not so quickly that you start making errors."

"Please do not search the answers online while you are completing the study; if you are unsure of an answer, please just make your best guess."

"Now you will see a series of trivia statements.

As a reminder, for each trivia statement you will be answering the following question:

Is this statement true or false?"

Experiment 1: An Analysis of the Magnitude of the ITE Across Secondary Task Condition Compared to the No Task Condition, Without Exclusions

In Experiment 1, we aimed to investigate two questions. Firstly, does including a secondary task impact the magnitude of the ITE? We found an ITE across secondary task conditions, however, there was no difference in the size of the ITE between No Task condition and Secondary Task conditions. Secondly, does participants' accuracy on the secondary task correlate with the magnitude of the ITE? We found that there was a positive association between the size of the ITE and overall task accuracy—the ITE magnitude increased with increasing accuracy on the secondary task.

Was there an ITE?

We ran a 2 (repetition: repeated, new) x 3 (secondary task: vowel-counting task, shoebox task, no task) repeated measures ANOVA on the mean truth ratings. There was a significant main effect of repetition on truth ratings, indicating an ITE was present, $F(1, 145) = 10.48, p < .002, partial\ eta\ squared = .07, 90\%\ CI$ [.02, .14]. There was no significant main effect of secondary task condition, $F(1, 145) = 0.73, p = .486, partial\ eta\ squared = .01, 90\%\ CI$ [.00, .04].

Did the size of the ITE differ depending on whether participants were given tasks?

There was no significant interaction between the ITE and secondary task condition, $F(1, 145) = 0.86, p = .426, partial\ eta\ squared = .01, 90\%\ CI$ [.00, .04]. Thus, there was no evidence of variation in the ITE between Vowel-counting Task, Shoebox Task or No Task conditions.

Was the magnitude of the ITE associated with participants' task accuracy?

We found a small but significant correlation between the magnitude of the ITE and overall accuracy, $r(98) = .20, p = .050, 95\%\ CI$ [.00, .40] (see Fig. 4). This finding demonstrates that as participants' accuracy on the secondary task increased, so did the size of the ITE.

Experiment 2: An Analysis of the Magnitude of the ITE Across Secondary Task Conditions Compared to the No Task Condition, Without Exclusions

In Experiment 2, we found a difference in the size of the ITE between the secondary task placement conditions (at encoding only, at test only, at encoding and at test, compared to no task). The ITE was largest in the *no task* condition compared to the other secondary task conditions. Further, we replicated the significant correlation between task accuracy and the size of the ITE.

Was there an ITE?

We ran a 2 (repetition: repeated, new) x 4 (secondary task placement condition: task at encoding, task at test, task at encoding and test, no task) repeated measures ANOVA on the mean truth ratings. There was a significant main effect of repetition on truth ratings, indicating an ITE was present, $F(1, 194) = 26.77, p < .001, partial\ eta\ squared = .12, 90\%\ CI\ [.06, .19]$. There was no significant main effect of secondary task placement condition, $F(3, 194) = 1.00, p = .395, partial\ eta\ squared = .02, 90\%\ CI\ [.00, .04]$.

Did the size of the ITE differ depending on when participants completed tasks?

There was a significant interaction between repetition and secondary task placement condition, $F(3, 194) = 3.70, p < .05, partial\ eta\ squared = .05, 90\%\ CI\ [.01, .10]$. This result shows that the size of the ITE differs across secondary task placement conditions: *task at encoding*, *task at test, task at encoding and test* and *no task.* In a follow-up paired samples t-test on the difference between mean truth ratings for repeated and new items, by secondary task placement condition, we found a significant ITE in the *no task*, *task at encoding* and *task at test* condition, but no ITE in the *task at encoding and test* condition. As evidenced by the raw mean differences within each condition and associated confidence intervals, the magnitude of the ITE was largest in the *no task* condition,($t(46) = 4.00, p < .001, raw\ mean\ difference\ = 0.60, 95\%\ CI\ [.30, .90]\ M_{Repeated} = 4.21, SD = 0.89;\ M_{New} = 3.61, SD = 0.72$). There was a significant, but smaller, ITE in the *task at encoding* condition, ($t\ (50) = 2.04, p = .047, raw\ mean\ difference\ = 0.25, 95\%\ CI\ [.00, .49]\ M_{Repeated} = 3.96, SD = 0.93;\ M_{New} = 3.72, SD = 0.87$), and the *task at test* condition, ($t\ (52) = 3.01, p = .004, raw\ mean\ difference\ = 0.40, 95\%\ CI\ [.13, .66]\ M_{Repeated} = 4.19, SD = 1.04;\ M_{New} = 3.82, SD = 0.99$). There was no significant ITE in the *task at encoding and test* condition, ($t\ (46) = 0.40, p = .688, raw\ mean\ difference\ = 0.03, 95\%\ CI\ [.11, .17]\ M_{Repeated} = 3.76, SD = 0.86;\ M_{New} = 3.73, SD = 0.88$).

Was the magnitude of the ITE associated with participants' task accuracy?

A correlational analysis revealed a significant correlation between the magnitude of the ITE and overall accuracy (%), $r(126) = .34, p < .001;\ 95\%\ CI\ [.17, .48]$. As in the two prior experiments, these results show that as participants' accuracy on the secondary task increases, the larger the ITE.

Experiment 3: An Analysis of the Magnitude of the ITE Across Secondary Task Conditions Compared to the No Task Condition, Without Exclusions

In Experiment 3, we found a difference in the size of the ITE between the secondary task placement conditions (at encoding only, at test only, at encoding and at test, compared to no task). The ITE was largest in the *no task* condition compared to the rest of the secondary task conditions. Again, we replicated a significant correlation between task accuracy and the size of the ITE.

Was there an ITE?

We ran a 2 (repetition: repeated, new) x 4 (secondary task placement condition: task at encoding, task at test, task at encoding and test, no task) repeated measures ANOVA on the mean truth ratings. There was a significant main effect of repetition on truth ratings, indicating an ITE was present, $F(1,162) = 103.72, p < .001, partial\ eta\ squared = .39, 90\%\ CI\ [.29, .47]$. There was no significant main effect of secondary task placement condition, $F(3,162) = 2.06, p = .107, partial\ eta\ squared = .04, 90\%\ CI\ [.00, .08]$.

Did the size of the ITE differ depending on when participants completed tasks?

There was a significant interaction between repetition and secondary task placement condition, $F(3,162) = 5.71, p = .001, partial\ eta\ squared = .10, 90\%\ CI\ [.03, .16]$. This result shows that the size of the ITE differs across secondary task placement conditions: *task at encoding*, *task at test, task at encoding and test* and *no task*. In a follow-up paired samples t-test on the difference between mean truth ratings for repeated and new items, by secondary task placement condition, we found a significant ITE in the *no task*, *task at encoding*, *task at test* condition, and *task at encoding and test* condition. As evidenced by the raw mean differences within each condition and associated confidence intervals, the magnitude of the ITE was largest in the *no task* condition,

$(t(39) = 6.40, p < .001, raw\ mean\ difference\ = 1.26, 95\%\ CI\ [.86, 1.65]\ M_{Repeated} = 4.59, SD = 1.08;\ M_{New} = 3.35, SD = 0.74)$. There was a significant, but smaller, ITE in the *task at encoding* condition, $(t\ (41) = 4.80, p < .001, raw\ mean\ difference\ = 0.76, 95\%\ CI\ [.00, .49]\ M_{Repeated} = 3.96, SD = 0.93;\ M_{New} = 3.72, SD = 0.87)$, and the *task at test* condition, $(t(39) = 5.23, p < .001, raw\ mean\ difference\ = 0.77, 95\%\ CI\ [.47, 1.07]\ M_{Repeated} = 4.13, SD = .94;\ M_{New} = 3.36, SD = 0.54)$. There was a significant ITE in the *task at encoding and test* condition, $(t\ (43) = 3.25, p < .05, raw\ mean\ difference\ = 0.36, 95\%\ CI\ [.14, .58]\ M_{Repeated} = 3.83, SD = 0.66;\ M_{New} = 3.47, SD = 0.68)$.

Was the magnitude of the ITE associated with participants' task accuracy?

A correlational analysis revealed a significant correlation between truth difference and overall accuracy (%), $r(104) = .24, p = .015;\ 95\%\ CI\ [.05, .41]$. As in the prior experiments, these results show that as participants' accuracy on the secondary task increases, the larger the ITE.

An Analysis of the Magnitude of the ITE Across Low vs. High Accuracy Groups in the Task Condition Compared to the No Task Condition

In Experiments 2 and 3, we found significant interaction effects between repetition and tangential task conditions (reported in the main manuscript), such that the magnitude of the ITE was larger in the No Task condition compared to the secondary task conditions where participants were given tasks. Specifically, in both Experiments 2 and 3, when looking at the raw mean difference (the size of the ITE) and the associated confidence intervals within each secondary task condition, the ITE appears to be largest in the No Task (control) condition. To further explore this finding, we did a median split on the overall task accuracy scores for each experiment and then ran a paired samples t-test on the difference between mean truth ratings for old vs new items by No Task condition vs. Task condition (conditions where tasks were given collapsed into one). See Table S5 for the results. Note, we also included findings from Experiment 1 despite there being no significant interaction effect between repetition and secondary task condition. This is because we want to investigate whether the pattern of results where the ITE was largest in the No Task condition was also present in Experiment 1.

5 Supplementary Table S5

Mean Differences in Truth Ratings for Old and New Items Across High vs. Low Task Accuracy Groups by No Task vs. Task Conditions in Experiments 1, 2, and 3

Condition	Overall Mean Accuracy	$Mean_{Old}$	$Mean_{New}$	Raw Mean Difference	SE	t-ratio	p	Upper 95%	Lower 95%
Experiment 1									
No Task		3.86	3.59	0.30	0.11	2.38	.02*	-0.04	-0.49
Task: High Accuracy	93.81	3.78	3.49	0.30	0.13	2.31	.02*	-0.04	-0.54
Task: Low Accuracy	64.86	4.16	4.15	0.01	0.08	0.12	.91	0.14	-0.16
Experiment 2									
No Task		4.22	3.62	0.61	0.15	3.97	.0003*	-0.30	-0.91
Task: High Accuracy	96.81	4.28	3.67	0.61	0.14	4.33	.0001*	-0.33	-0.89
Task: Low Accuracy	51.79	3.85	3.80	0.05	0.06	0.74	.465	0.08	-0.18
Experiment 3									
No Task		4.59	3.33	1.26	0.20	6.40	.0001*	-0.86	-1.65
Task: High Accuracy	100.00	4.05	3.29	0.76	0.12	6.09	.0001*	-0.51	-1.01
Task: Low Accuracy	82.73	4.06	3.36	0.70	0.13	5.18	.0001*	-0.43	-0.97

Note. Overall accuracy scores are percentages (max =100%) and "*" indicate the difference in mean truth ratings for old items and new items were significant.

A Correlational Analysis of the Magnitude of the ITE Across Low vs. High Accuracy Groups in the Task Condition of Experiment 1, 2 and 3

As mentioned in the main text, when we split participants in all the task conditions by high vs. low accuracy, those in the high accuracy group show a similar ITE to the No Task condition. This pattern of results was present in both Experiment 1 and 2, but were only directional in Experiment 3. Taken together, this finding suggests that the correlation may be driven by the low accuracy participants who show a significantly smaller ITE when compared to the No Task condition. Indeed, this was supported in our further analyses. We ran a correlational analysis between accuracy and truth difference scores (the magnitude of the ITE) for the low accuracy and high accuracy group. For Experiment 2 and 3, we found a significant positive correlation between task accuracy and the magnitude of the ITE in the low accuracy group but not the high accuracy group. We report the results below.

Was the magnitude of the ITE associated with participants' task accuracy in the Low Accuracy group?

Experiment 1: A correlational analysis revealed a non-significant correlation between the magnitude of the ITE and overall accuracy, $r(37) = .16, p = .359, 95\%\ CI$ [.18, .46]. These results suggested no evidence that, within the low accuracy group, participants' overall accuracy on the secondary task was associated with the size of the ITE.

Experiment 2: A correlational analysis revealed a significant positive correlation between the magnitude of the ITE and overall accuracy, $r(56) = .29, p = .033, 95\%\ CI$ [.02, .51]. These results show that, within the low accuracy group, as participants' overall accuracy on the secondary task increases, the ITE increases.

Experiment 3: A correlational analysis revealed a significant positive correlation between the magnitude of the ITE and overall accuracy, $r(54) = .33, p = .014, 95\%\ CI$ [.07, .55]. These results show that, within the low accuracy group, as participants' overall accuracy on the secondary task increases, the ITE increases.

Was the magnitude of the ITE associated with participants' task accuracy in the High Accuracy group?

Experiment 1: A correlational analysis revealed a non-significant correlation between the magnitude of the ITE and overall accuracy, $r(50) = .19, p = .184, 95\%\ CI$ [.09, .45]. These results suggested no evidence that, within the high accuracy group, participants' overall accuracy on the secondary task was associated with the size of the ITE.

Experiment 2: A correlational analysis revealed no significant correlation between the magnitude of the ITE and overall accuracy, $r(58) = .10, p = .441, 95\%\ CI$ [−.16, .35]. These results show that, within the high accuracy group, participants' overall accuracy on the secondary task was not associated with the magnitude of the ITE.

Experiment 3: A correlational analysis revealed no significant correlation between the magnitude of the ITE and overall accuracy, $r(47) = 0, p = 1.00, 95\%\ CI\ [-.29, .40]$. These results show that, within the high accuracy group, participants' overall accuracy on the secondary task was not associated with the magnitude of the ITE.
